# *In silico* analyses of dystrophin Dp40 cellular distribution, nuclear export signals and structure modeling

**DOI:** 10.1016/j.dib.2015.06.007

**Published:** 2015-06-25

**Authors:** Alejandro Martínez-Herrera, Jorge Aragón, Rosa Ma. Bermúdez-Cruz, Ma. Luisa Bazán, Gabriela Soid-Raggi, Víctor Ceja, Andrea Santos Coy-Arechavaleta, Víctor Alemán, Francisco Depardón, Cecilia Montañez

**Affiliations:** a Departamento de Genética y Biología Molecular, Centro de Investigación y de Estudios Avanzados del Instituto Politécnico Nacional, México, D. F., Mexico; b Departamento de Fisiología, Biofísica y Neurociencias, Centro de Investigación y de Estudios Avanzados del Instituto Politécnico Nacional, México, D. F., Mexico

**Keywords:** *In silico*, Dystrophin Dp40, Cellular distribution

## Abstract

Dystrophin Dp40 is the shortest protein encoded by the DMD (Duchenne muscular dystrophy) gene. This protein is unique since it lacks the C-terminal end of dystrophins. In this data article, we describe the subcellular localization, nuclear export signals and the three-dimensional structure modeling of putative Dp40 proteins using bioinformatics tools. The Dp40 wild type protein was predicted as a cytoplasmic protein while the Dp40n4 was predicted to be nuclear. Changes L93P and L170P are involved in the nuclear localization of Dp40n4 protein. A close analysis of Dp40 protein scored that amino acids ^93^LEQEHNNLV^101^ and ^168^LLLHDSIQI^176^ could function as NES sequences and the scores are lost in Dp40n4. In addition, the changes L93/170P modify the tertiary structure of putative Dp40 mutants. The analysis showed that changes of residues 93 and 170 from leucine to proline allow the nuclear localization of Dp40 proteins. The data described here are related to the research article entitled “EF-hand domains are involved in the differential cellular distribution of dystrophin Dp40” (J. Aragón et al. Neurosci. Lett. 600 (2015) 115–120) [1].

Specifications table

**Table:** 

Subject area	Biology
More specific subject area	Dystrophin bioinformatics analyses
Type of data	Table, image
How data was acquired	*In silico* analyses: PSORT II program (http://www.genscript.com/psort/psort2.html) was used to predict the protein localizations; NetNES 1.1 server (http://www.cbs.dtu.dk/services/NetNES/) to search nuclear export signal; and SWISS-MODEL and I-TASSER programs (http://zhanglab.ccmb.med.umich.edu/I-TASSER/) for modeling protein structures. The comparative modeling was made in: http://www.pymol.org/funding.html
Data format	Analyzed
Experimental factors	N/A
Experimental features	N/A
Data source location	Departamento de Genética y Biología Molecular, Centro de Investigación y de Estudios Avanzados del Instituto Politécnico Nacional, México, D. F., México.
Data accessibility	Dp40 mRNA sequence, accession number: KF154977, http://www.ncbi.nlm.nih.gov/nuccore/KF154977. Dp40 protein sequence, http://www.ncbi.nlm.nih.gov/protein/543869031.

Value of the data

• Bioinformatics tools permit to search putative dystrophin Dp40 protein domains and/or functions;
• PSORT II program is an alternative tool to screen for the subcellular localization of Dp40 proteins;
• NetNES 1.1 server allows to identify putative nuclear export signals of dystrophin Dp40;
• Comparative modeling analysis between Dp40 and Dp40 mutants identify differences in protein structure.

## 1 Data, experimental design, materials and methods

### 1.1 Prediction of the subcellular localization of putative Dp40 and mutant proteins

To predict the subcellular localization, the amino acids sequence of Dp40 protein (Protein ID: AGV74356.1) was analyzed using PSORT II software (http://www.genscript.com/psort/psort2.html) [2]. The Dp40 protein was predicted as a cytoplasmic protein while the predicted localization of Dp40n4, carrying changes L93P and L170P (L93/170P) into the EF1 and EF2 hand domains, was nuclear (Table 1). Additional changes are present in Dp40n4 (M288T and D303G); however, none of these changes modified the predictions of the subcellular localization and NES score. Interestingly, the replacement of proline to leucine residues in Dp40n4, either Dp40n4-P93L, Dp40n4-P170L or Dp40n4-P93/170L, was scored as cytoplasmic (Table 1). In addition, the replacement of leucines 93 and 170 to proline residues in Dp40 (Dp40-L93/170P), was predicted to have a nuclear localization (Table 1) which was confirmed by site-directed mutagenesis [1].

### 1.2 Identification of putative nuclear export signals in Dp40 amino acid sequence

**Fig. 1:**
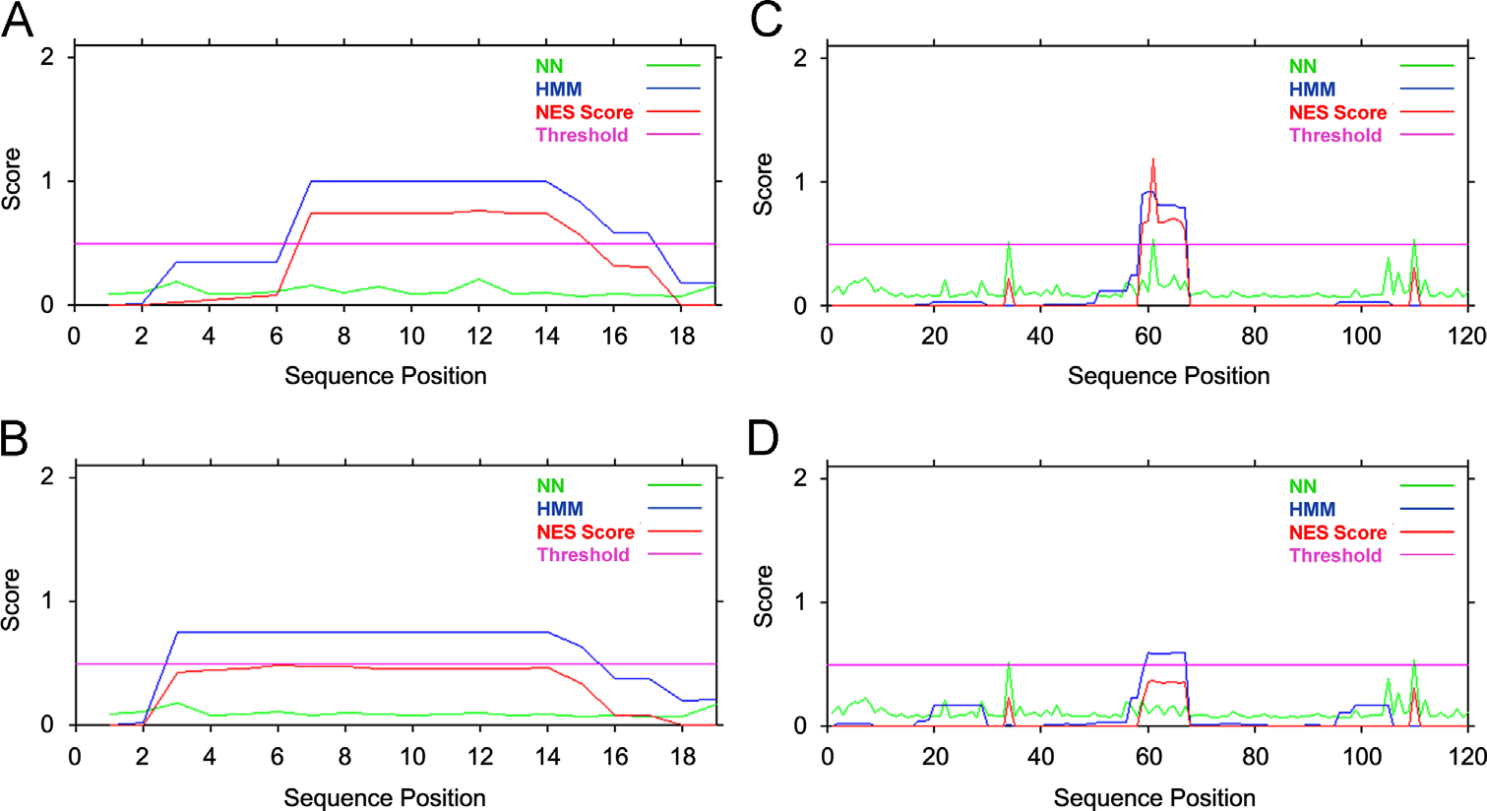
Prediction of putative nuclear export signals in Dp40 proteins. Analyses of Dp40 amino acids were performed using the NetNES 1.1 server [3]. (A) Analysis of Dp40 amino acids T87 to C106. (B) Analysis of Dp40 amino acids C110 to L230. (C) Analysis of Dp40n4 and Dp40-L93/170P amino acids T87 to C106. (D) Analysis of Dp40n4 and Dp40-L93/170P amino acids C110 to L230. Putative nuclear export signals (NES score), amino acids ^93^LEQEHNNLV^101^ and ^168^LLLHDSIQI^176^, were identified in Dp40 (A and B, respectively) and values of NES score were lost in Dp40n4 and Dp40-L93/170P (C and D, respectively). NN, Neural Network. HMM, Hidden Markov Model.

To identify possible nuclear export signals (NES), a close analysis of Dp40 and Dp40n4 amino acid sequences was carried out using the NetNES 1.1 server (http://www.cbs.dtu.dk/services/NetNES/) [3]. Analyses of Dp40 amino acids from T87 to C106 and C110 to L230 scored that amino acids ^93^LEQEHNNLV^101^ and ^168^LLLHDSIQI^176^ could function as NES sequences (Fig. 1A and B, respectively) and the NES score was lost when Dp40n4 and Dp40-L93/170P amino acids (^93^PEQEHNNLV^101^ and ^168^LLPHDSIQI^176^; changes L93P and L170P are underlined) were analyzed (Fig. 1C and D, respectively).

### 1.3 Comparative modeling analyses between Dp40 and mutant proteins

**Fig. 2:**
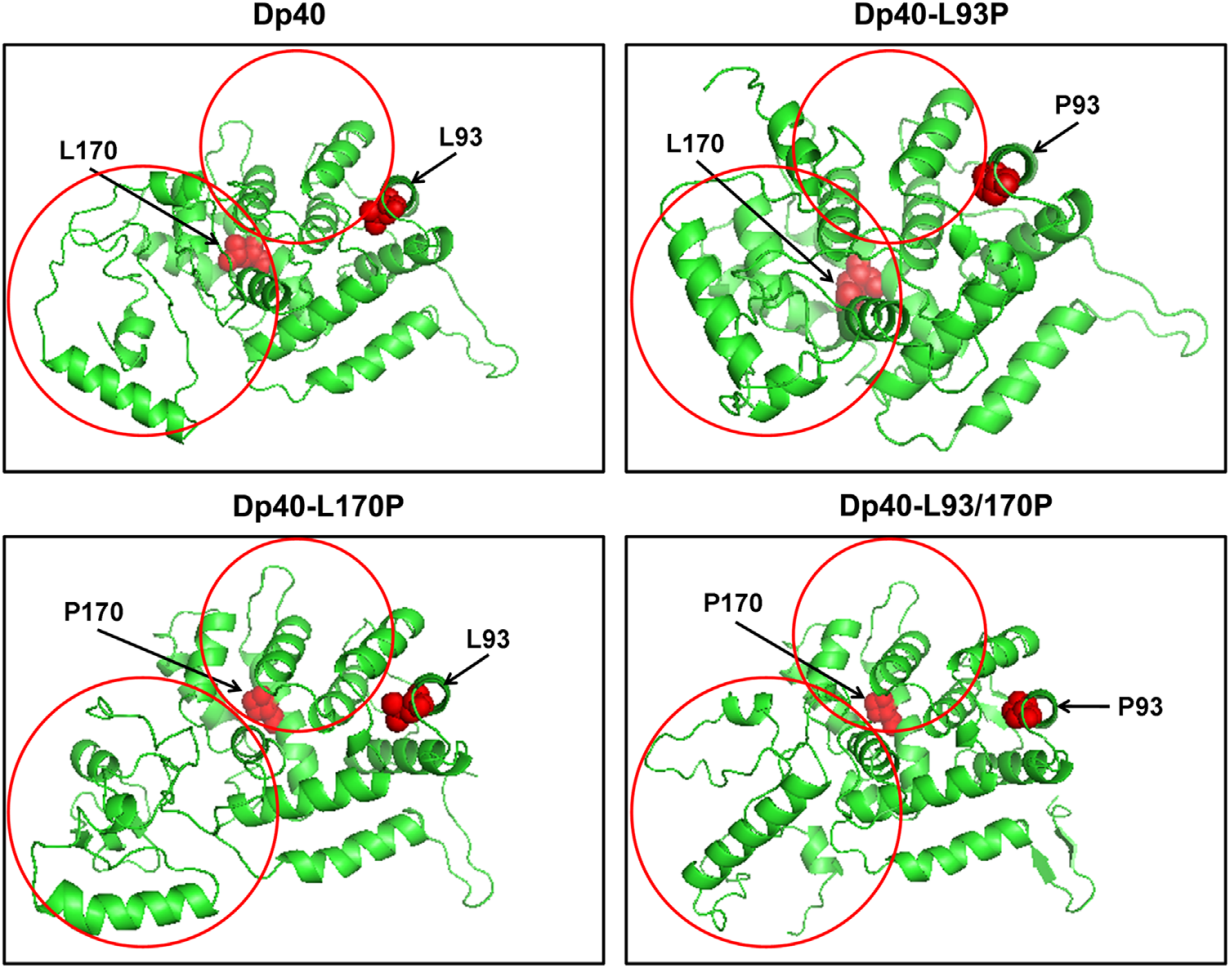
Comparative modeling of Dp40, Dp40-L93P, Dp40-L170P and Dp40-L93/170P proteins. Structure modeling was carried out using SWISS-MODEL and I-TASSER programs [4,5]. Circles indicate differences between Dp40 and Dp40 mutants. Arrows indicate leucine and/or proline residues 93 and 170 (red).

To identify whether mutations L93P and L170P modify the tertiary structure of Dp40 proteins, structure modeling of Dp40 and Dp40 mutants were analyzed using SWISS-MODEL and I-TASSER programs [4,5]. Fig. 2 shows the tertiary structure modeling of putative Dp40 proteins. Differences are observed between Dp40 and Dp40 mutants as well as among Dp40 mutants.

## Conflict of interest

The authors declare that there is no conflict of interest.

## Figures and Tables

**Table 1:** Prediction of the subcellular localization (%) of putative Dp40 proteins.

Protein	Outside the nucleus^a^	Nuclear
Dp40	69.6	30.4
Dp40-L93P	69.6	30.4
Dp40-L170P	69.6	30.4
Dp40-L93/170P	39.1	60.9
Dp40n4	39.1	60.9
Dp40n4-P93L	69.6	30.4
Dp40n4-P170L	69.6	30.4
Dp40n4-P93/170L	69.6	30.4

a Includes cytoplasmic, mitochondrial and peroxisomal localization.
